# LncRNA MEG3 inhibits cell proliferation and induces apoptosis in laryngeal cancer via miR‐23a/APAF‐1 axis

**DOI:** 10.1111/jcmm.14549

**Published:** 2019-07-21

**Authors:** Xiaowen Zhang, Nan Wu, Jin Wang, Zhijie Li

**Affiliations:** ^1^ Medical Research Center Shengjing Hospital of China Medical University Shenyang China; ^2^ Key Laboratory of Research and Application of Animal Model for Environmental and Metabolic Diseases Shenyang China; ^3^ The Central Laboratory of the First Affiliated Hospital of China Medical University Shenyang China; ^4^ The ENT Department Shengjing Hospital of China Medical University Shenyang China

**Keywords:** apoptotic protease activating factor‐1, competitive endogenous RNA, laryngeal cancer, maternally expressed gene 3, miRNA‐23a

## Abstract

Long non‐coding RNA (LncRNA) MEG3 serves a regulatory role in the progression of several types of cancer, but the role of MEG3 in laryngeal cancer is still unknown. The aim of this study was to explore the regulatory role and mechanism of MEG3 in laryngeal cancer. MEG3 expression in 50 laryngeal cancer tissue samples was detected by reverse transcription‐quantitative polymerase chain reaction (RT‐qPCR). The effects of MEG3 overexpression on laryngeal cancer cells were investigated in vitro and in vivo. The mechanism of competitive endogenous RNA (ceRNA) was validated through luciferase reporter assay, RT‐qPCR and Western blotting. MEG3 was down‐regulated in laryngeal cancer tissues, and the low MEG3 expression was associated with advanced clinical stage. Additionally, MEG3 overexpression inhibited the proliferation and induced the apoptosis of laryngeal cancer cells in vitro and in vivo. Particularly, MEG3 bound to miR‐23a specifically and a reciprocal negative regulation existed between miR‐23a and MEG3. Moreover, MEG3 up‐regulated apoptotic protease activating factor‐1 (APAF‐1), a known miR‐23a's target, thereby leading to the activation of caspase‐9 and caspase‐3. Meanwhile, these activated effects were rescued by miR‐23a overexpression. In conclusion, the present study demonstrated that MEG3 functions as a novel tumour suppressive LncRNA in laryngeal cancer for the first time. Furthermore, MEG3 may act as a ceRNA to regulate APAF‐1 expression by competitive binding to miR‐23a, thereby regulating the progression of laryngeal cancer.

## 1 INTRODUCTION

Laryngeal cancer is the highest incidence of head and neck cancer and probably accounts for 1%～5% cancer incidence worldwide.^1^ Smoking and alcohol are considered as common major risk factors for laryngeal cancer.^2^ Moreover, some researchers have found that people with a family history of cancer are also susceptible to laryngeal cancer, which implicates that genetic factors serve an important role in the occurrence and progression of laryngeal cancer.^3^ Growing evidence has demonstrated that some tumour suppressor genes, such as CDKN2A, TP53 and CTNNA2, are down‐regulated or inactivated in laryngeal cancer tissues and contributed to the tumorigenesis of laryngeal cancer.^4^, ^5^ Recent studies have found that long non‐coding RNAs (LncRNAs), a member of non‐coding RNA gene family, are dysregulated in laryngeal cancer tissues.^6^, ^7^ More interestingly, several LncRNAs, such as HOTAIR,^8^ NEAT1^9^ and H19,^10^ function as oncogenes to promote the tumorigenesis of laryngeal cancer. Therefore, identifying the role of LncRNAs in laryngeal cancer may bring a novel insight into the occurrence and development of laryngeal cancer.

Maternally expressed gene 3 (MEG3), located in human chromosome 14q32.3 region, has been identified as a LncRNA because its transcript lacks a significant open reading frame. MEG3 is found to be down‐regulated in cancer tissues and lost in some cancer cells.^11^ Moreover, MEG3 is characterized as a tumour suppressor as growing evidence that overexpression of MEG3 inhibits tumour cell growth, invasion and metastasis, induces tumour cell apoptosis and enhances tumour cell chemosensitivity.^12^ However, the role of MEG3 in laryngeal cancer remains unknown. Furthermore, recent study has demonstrated that MEG3 could modulate endogenous miRNAs available for binding to their target mRNAs acting as miRNA sponges, also called competing endogenous RNA (ceRNA), thereby leading to the repression of these mRNAs.^13^ For instance, MEG3 could modulate Bcl‐2 by competitively binding to miR‐181 to regulate gastric carcinogenesis.^14^ Additionally, we predicted that MEG3 could bind to miR‐23a specifically using an online bioinformatics tool (LncBase Predicted v.2)^15^ and our previous study has demonstrated that miR‐23a promotes the laryngeal cancer cell proliferation and induces the apoptosis by targeting apoptotic protease activating factor‐1 (APAF‐1).^16^ Therefore, we have been suggested that MEG3 regulates APAF‐1 expression by functioning as a ceRNA to competitively bind to miR‐23a, thereby modulating the progression of laryngeal cancer.

In the present study, the result showed that the expression of MEG3 was down‐regulated in laryngeal cancer tissues. Overexpression of MEG3 inhibited the growth, proliferation and induced the apoptosis of laryngeal cancer cell in vitro and in vivo. Mechanistically, MEG3 acted as a ceRNA to regulate APAF‐1 expression via competitively binding to miR‐23a.

## 2 MATERIALS AND METHODS

### 2.1 Laryngeal cancer patients and tissue samples

A total of 50 pairs of tumour and adjacent normal tissues were collected from laryngeal cancer patients undergoing laryngectomy in the Otolaryngology Department of the No. 463 Hospital of PLA, consisting of 43 males and 7 females with a median age of 60 years (range, 39‐82 years). The clinicopathological characteristics of 50 laryngeal cancer patients were summarized in Table 1. Informed consent was obtained from each patient before this study. The histopathological diagnosis for each sample was performed by two pathologists independently. Tumour stages were determined according to the American Joint Committee on Cancer (AJCC) tumour‐node‐metastasis (TNM) staging criteria. The research protocols were approved by the Ethics Committee of Shengjing Hospital of China Medical University.

**Table 1:** The clinicopathological characteristics of 50 laryngeal cancer patients

Features	No. of cases (%)
Age	
<60	23 (46)
≥60	27 (54)
Gender	
Male	43 (86)
Female	7 (14)
Smoking	
Non‐smokers	7 (14)
Current smokers	43 (86)
Drinking	
Drinker	35 (70)
Non‐drinker	15 (30)
Differentiation	
Well	15 (30)
Moderate	28 (56)
Poor	7 (14)
Lymph node	
Negative	32 (64)
Positive	18 (36)
Tumour extension (pT)	
T1	8 (16)
T2	14 (28)
T3	15 (30)
T4	13 (26)
Clinical stage	
Ⅰ	5 (10)
Ⅱ	11 (22)
Ⅲ	31 (62)
Ⅳ	3 (6)

### 2.2 Cell cultures and cell transfection

Hep‐2 and AMC‐HN‐8 human laryngeal cancer cell lines were obtained from Cell Biology Institute of Shanghai, Chinese Academy of Science and American Type Culture Collection (ATCC), respectively. Cells were cultured in RPMI 1640 medium added with 10% foetal bovine serum (FBS), 100 U/mL penicillin and 100 µg/mL streptomycin at 37°C in a humidified incubator with 5% CO_2_.

Maternally expressed gene 3 expression plasmid (pcDNA3.1‐MEG3) and empty pcDNA3.1 vectors were gifted from Prof. Ka‐Fai To (Department of Anatomical and Cellular Pathology, Prince of Wales Hospital, The Chinese University of Hong Kong). The details for construction of MEG3 plasmid were described in previous research.^11^ pcDNA3.1‐MEG3 and empty pcDNA3.1 vectors at a final concentration of 2 μg/mL were transfected into cells using Lipo2000 (Invitrogen) according to the manufacturer's instructions. miR‐23a mimic and negative control (mimic NC) designed and synthesized by GenePharma Co Ltd at a final concentration of 100 nmol were transfected into cells using Lipo2000 according to the manufacturer's instructions. The cells were harvested and processed for further analysis after 24 or 48 hours of transfection.

### 2.3 RNA isolation and reverse transcription‐quantitative polymerase chain reaction (RT‐qPCR)

Total RNA was extracted from tissues and cells with TRIzol reagent (Invitrogen). To detect MEG3 and mRNA expression, PrimeScript RT Reagent Kit with gDNA Eraser (TaKaRa) and SYBR Premix Ex Taq II (TaKaRa) were used for reverse transcription and quantitative PCR according to the manufacturer's protocol. GAPDH was used as an internal control. To detect miR‐23a expression, Mir‐X™ miRNA First Strand Synthesis Kit and Mir‐X™ miRNA RT‐qPCR SYBR^®^ Kit were used for reverse transcription and quantitative PCR according to the manufacturer's protocol. U6 was used as an internal control. All oligonucleotide primers were synthesized by Jinsirui Biotech Co Ltd as follows: MEG3 forward 5′‐CCTGCTGCCATCTACACCTC‐3′; MEG3 reverse 5′‐CCTCTTCATCCTTTGCCATCCTGG‐3′; APAF‐1 forward 5′‐GAAGAGAACCTGGGAGTAGATAAGG‐3′; APAF‐1 reverse 5′‐GTCGTAGCAAACCACCAAGC‐3′; miR‐23a forward 5′‐ATCACATTGCCAGGGATTTCC‐3′. The relative expression was analysed using 2^−ΔΔCt^ method.

### 2.4 Ethynyldeoxyuridine (EdU) assay

Cell proliferation was detected by EdU assay. Specifically, the cells were cultured in 96‐well plates at a density of 0.6 × 10^4^ cells/well. At 46 hours post‐transfection, cells were incubated with 20 μmol/L EdU labelling media (RiboBio) for 2 hours at 37°C with 5% CO_2_. After fixation with 4% paraformaldehyde for 30 minutes, the cells were stained with anti‐EdU working solution. The percentage of EdU‐positive cells was calculated after fluorescence microscopy analysis.

### 2.5 CCK‐8 assay

Cell proliferation was measured by CCK‐8 assay using Cell Counting Kit‐8 (KeyGEN Biotech) in accordance with the manufacturer's instructions. Hep‐2 and AMC‐HN‐8 cells (3 × 10^3^ cells/well) were seeded into 96‐well plates and incubated with CCK‐8 solution (10 µL/well) at 37°C with 5% CO_2_ for 4 hours. The optical density (OD) value of each well was detected using a microplate reader set at the absorbance of 450 nm. The changes in cell proliferation were determined for 5 consecutive days.

### 2.6 Colony formation assay

After 24 hours of transfection, cells (3 × 10^3^ cells/well) were seeded into 6‐well plates and incubated in RPMI 1640 (GIBCO) with 10% FBS. After 9 days, colonies were harvested and fixed with methanol for 30 minutes, stained with haematoxylin for 20 minutes and photographed by a digital camera (Nikon).

### 2.7 Cell apoptosis measured by flow cytometry

The cell apoptosis was measured by flow cytometry using Annexin V‐FITC/PI Apoptosis Detection Kit (KeyGEN Biotech) according to the manufacturer's instructions. Data collection and analysis were performed using flow cytometry analyser (FACSCalibur).

### 2.8 Hoechst staining assay

Cells were cultured in 24‐well plates that were plated with glass slides in advance and transfected with pcDNA‐MEG3 and empty vector. After 48 hours of transfection, cells were fixed with 4% paraformaldehyde for 30 minutes and then incubated with 0.5 mL Hoechst 33342 solution (Wanlei, Shenyang, China) for 5 minutes at room temperature. After that, cells were washed twice with PBS and the changes in nuclear morphology were observed under fluorescence microscopy with 350 nm excitation and 461 nm emission. The number of Hoechst‐positive nuclei per optical field (at least 10 fields) was counted in three independent experiments and further calculated the ratio of apoptotic cell.

### 2.9 In vivo tumorigenicity assay

The animal experiment was approved by the Animal Ethics Committee of Shengjing Hospital of China Medical University, and all care and use of animals were in accordance with the Guide for the Care and Use of Laboratory Animals of the National Institutes of Health. To establish tumour xenograft model, 1 × 10^7^ Hep‐2 cells transfected with MEG3 plasmid and empty vector were injected subcutaneously into the right flank of eight 6‐week‐old Balb/c nude mice obtained from Changsheng Biological Technology Co Ltd. Tumour growth was examined every three days with callipers, and tumour volumes were calculated according to the following formula: ½ × length × width^2^. After 15 days of injection, the mice were killed under sodium pentobarbital anaesthesia and the xenograft tumours were resected and weighed.

### 2.10 Immunohistochemistry (IHC) analysis

The xenograft tumour tissues were fixed in 4% polyoxymethylene, embedded in paraffin and made into 3.5‐μm‐thick sections. The sections were immersed in 0.01 mol/L citrate buffer (pH = 6.0) and treated with microwaves for antigen retrieval. After inactivation of endogenous peroxidase activity with 3% H_2_O_2_ and blocking of nonspecific reactions with 10% goat serum, the sections were incubated with Ki67 (Santa Cruz Biotechnology) and cleaved caspase‐3 (Abcam) antibody overnight at 4°C, followed by incubation with an HRP‐conjugated secondary antibody for 1 hour at room temperature. The sections were stained with the 3,3‐diaminobenzidine (DAB) solution (Santa Cruz Biotechnology) and counterstained with haematoxylin.

### 2.11 Luciferase activity assay

The fragment of MEG3 containing the predicted miR‐23a binding sites was amplified by PCR and thereby constructing pcDNA‐MEG3 Wt (GenePharma Co Ltd). The pcDNA‐MEG3 Mut was synthesized by mutating the target sequences in MEG3 (GenePharma Co Ltd). Then, pcDNA‐MEG3 Wt and pcDNA‐MEG3 Mut were, respectively, cloned into GV272 luciferase reporter vectors (GenePharma Co Ltd), thereby constructing GV272‐MEG3‐Wt and GV272‐MEG3‐Mut. The cells were seeded in 24‐well plate and transfected with GV272‐MEG3‐Wt and GV272‐MEG3‐Mut together with miR‐23a mimic or mimic NC. At 48 hours post‐transfection, luciferase activity was analysed using the Dual‐Glo Luciferase Assay System (Promega) according to the manufacturer's instructions.

### 2.12 Western blotting

The tissues and cells were smashed and homogenized with RIPA (Beyotime). After centrifugation, the supernatant containing protein was collected. Protein concentrations were measured by using the BCA protein concentration Kit (Beyotime). After 50 μg protein was denatured at 100˚C for 10 minutes, SDS‐PAGE electrophoresis was performed for protein separation, and then, the protein was transferred to PVDF membrane. The PVDF membrane was blocked by 5% skim milk for 1 hour and then incubated with specific primary antibodies, including anti‐APAF‐1 (1:1000; Abcam), anti‐cleaved caspase‐9 (1:1000; Abcam), anti‐cleaved caspase‐3 (1:1000; Abcam) and anti‐GAPDH (1:1000; Zhongshan Jinqiao Biotechnology), at 4°C overnight. The PVDF membrane was incubated with horseradish peroxidase labelled as goat anti‐rabbit or goat anti‐mouse immunoglobulin G (1:4000; EarthOx) at room temperature for 30 minutes. Detection of protein band was performed by using an enhanced chemiluminescence (ECL) for Western blotting kit (Beyotime) according to the manufacturer's instructions. Relative densitometry was calculated by using Image J2x analysis software.

### 2.13 Statistical analysis

The SPSS 17.0 software was used for statistical analysis. Data were expressed as the mean ± SEM. Difference between two groups was evaluated using Student's *t* test. Difference within three or more groups was evaluated using one‐way analysis of variance (ANOVA), and if the difference was significant, multiple comparison analysis was further performed using Fisher's least significant difference (LSD) test. All *P* values < .05 were considered statistically significant.

## 3 RESULTS

### 3.1 Expression of MEG3 in laryngeal cancer tissues

**Figure 1:**
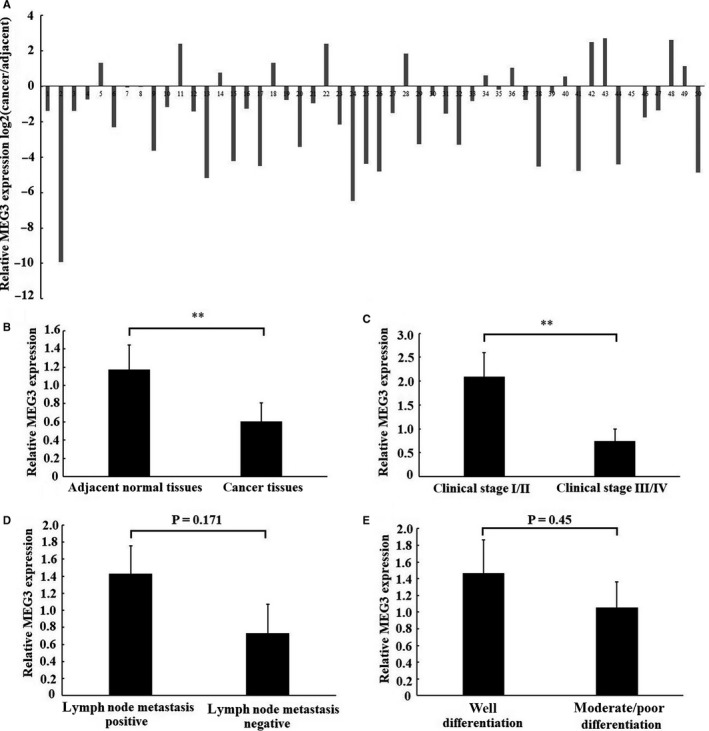
Expression of MEG3 in laryngeal cancer tissues. The relative expression of MEG3 was examined using RT‐qPCR in 50 patients with laryngeal cancer. A, The relative MEG3 expression was detected in each of laryngeal cancer tissues and matched normal tissues. Data were presented as fold change of laryngeal cancer tissues relative to adjacent normal regions (B) Relative MEG3 expression level in laryngeal cancer tissues and adjacent normal regions. (C), (D) and (E) the statistical analysis of the association between MEG3 expression and clinical stages, lymph node metastasis and differentiation. Data were presented as mean ± SEM, **P < .01

To explore the expression pattern of MEG3 in laryngeal cancer tissues, 50 pairs of laryngeal cancer samples were analysed by RT‐qPCR. The result showed that MEG3 expression was significantly down‐regulated in laryngeal cancer tissues compared with adjacent normal tissues. Specifically, MEG3 expression was decreased in 74% (37 out of 50) detected sample (Figure 1A,B). Moreover, we analysed the association between MEG3 expression and clinical features. The findings indicated that the low MEG3 expression was associated with advanced clinical stage of laryngeal cancer patients (Figure 1C). However, there was no statistical difference in the MEG3 expression between lymph node metastasis group and non‐lymph node metastasis group (Figure 1D) as well as between well differentiation group and moderate/poor differentiation group (Figure 1E).

### 3.2 MEG3 regulated the proliferation and apoptosis of laryngeal cancer cells

**Figure 2:**
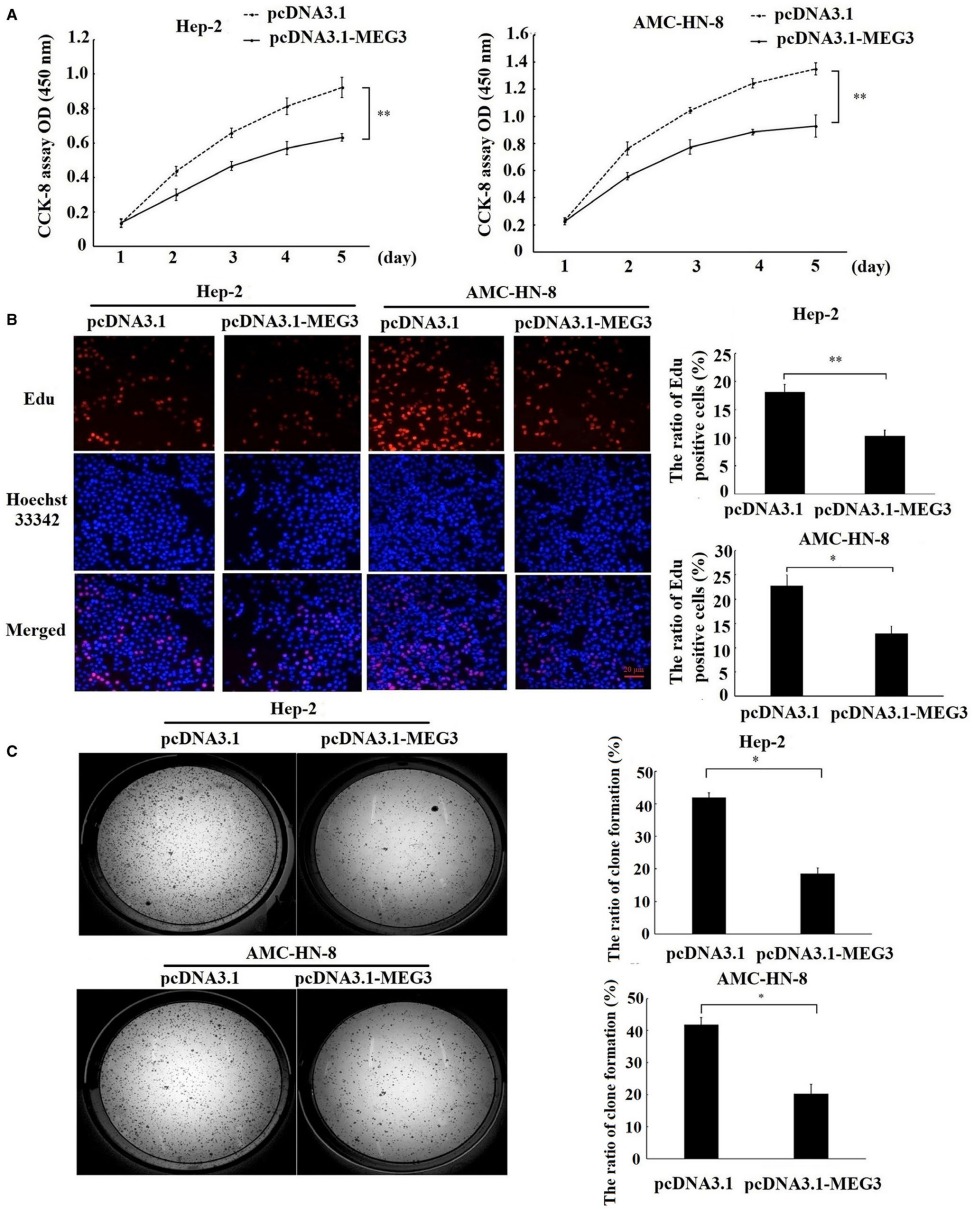
Effects of MEG3 on the proliferation of laryngeal cancer cells in vitro. Hep‐2 and AMC‐HN‐8 cells were transfected with MEG3 plasmid (pcDNA3.1‐MEG3) and empty vectors (pcDNA3.1). A, CCK‐8 assay was performed to measure the cell proliferation for 5 consecutive days. B, EdU assay was performed to evaluate the cell proliferation. Magnification: ×200, scale bars: 20 μm. C, Clone formation assay was performed to detect the ability of clone formation in Hep‐2 and AMC‐HN‐8 cells. The data were obtained from three independent experiments and presented as mean ± SEM, *P < .05.**P < .01

**Figure 3:**
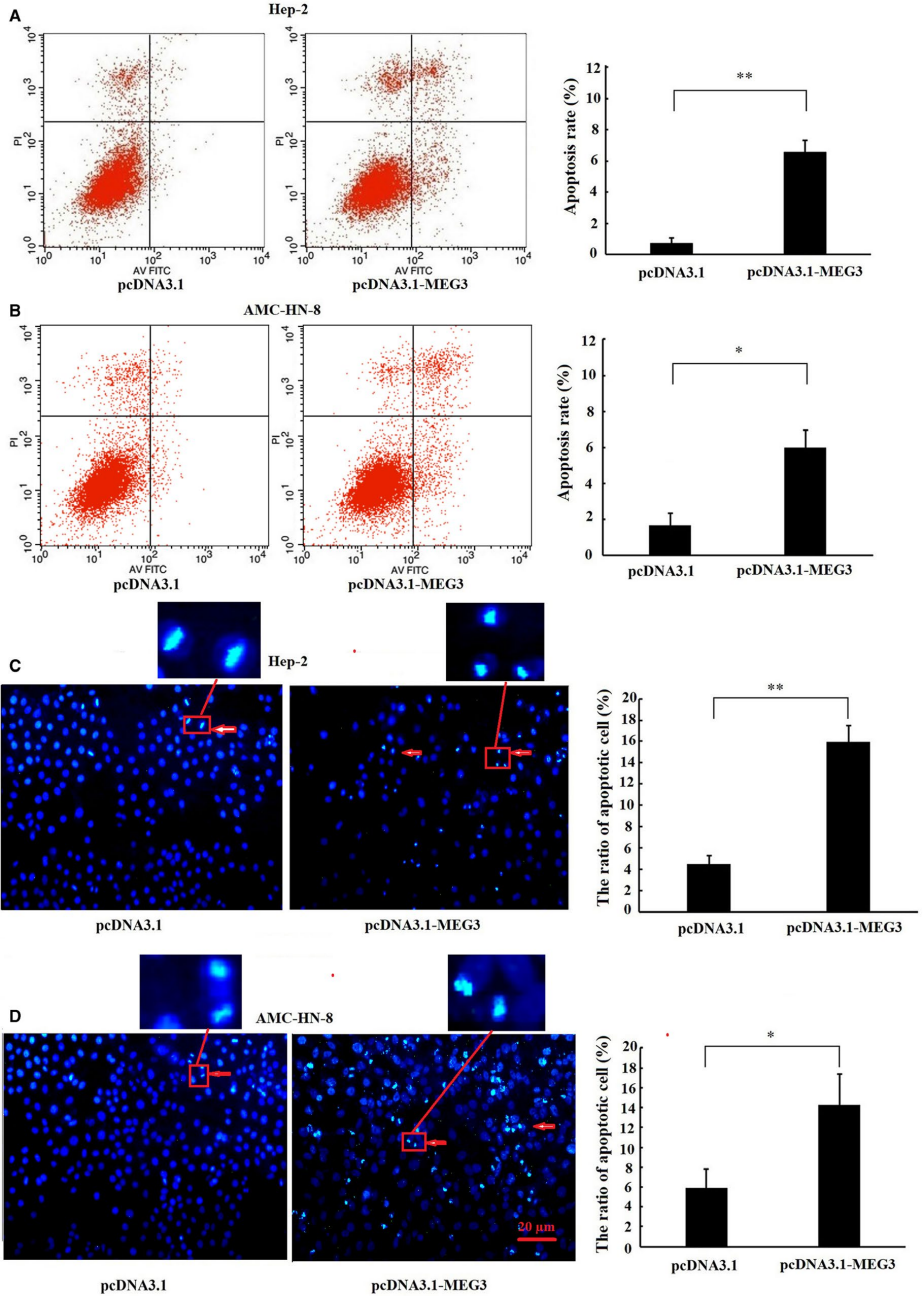
Effects of MEG3 on the apoptosis of laryngeal cancer cells. Hep‐2 and AMC‐HN‐8 cells were transfected with MEG3 plasmid (pcDNA3.1‐MEG3) and empty vectors (pcDNA3.1). Flow cytometry was employed to detect the apoptotic rates of cells in Hep‐2 (A) and AMC‐HN‐8 cells (B), cells in the lower right quadrant represent apoptosis cells. Hoechst staining assay was performed to assess cell apoptosis in Hep‐2 (C) and AMC‐HN‐8 cells (D). Hoechst‐positive nuclei (apoptotic nuclei) showed dense and high‐density fluorescence, as indicated by arrows, and the percentage of Hoechst‐positive nuclei per optical field (at least 10 fields) was counted. Magnification: ×200, scale bars: 20 μm. The data were obtained from three independent experiments and presented as mean ± SEM, *P < .05, **P < .01

To investigate the effect of MEG3 on the proliferation of laryngeal cancer cells, CCK‐8 assay, EdU assay and colony formation assay were employed. The result of CCK‐8 assay showed that Hep‐2 and AMC‐HN‐8 cells transfected with MEG3 plasmid displayed a lower ratio of proliferation as compared with cells transfected with empty vector (Figure 2A), which suggested that overexpression of MEG3 inhibited the proliferation of laryngeal cancer cells in vitro. In addition, EdU assay (Figure 2B) and colony formation assay (Figure 2C) showed similar results with respect to proliferation. Next, we explored the regulation of MEG3 on the apoptosis of laryngeal cancer cells using flow cytometry and Hoechst 33342 staining. The results showed that Hep‐2 (Figure 3A,C) and AMC‐HN‐8 cells (Figure 3B,D) transfected with MEG3 plasmid displayed a higher apoptosis rate as compared with cells transfected with empty vector, which suggesting that the apoptosis of laryngeal cancer cells was induced by up‐regulation of MEG3. Taken together, our results suggest that MEG3 inhibits the proliferation and induces the apoptosis of laryngeal cancer cells in vitro.

### 3.3 MEG3 regulated the growth, proliferation and apoptosis of laryngeal cancer cells in vivo

**Figure 4:**
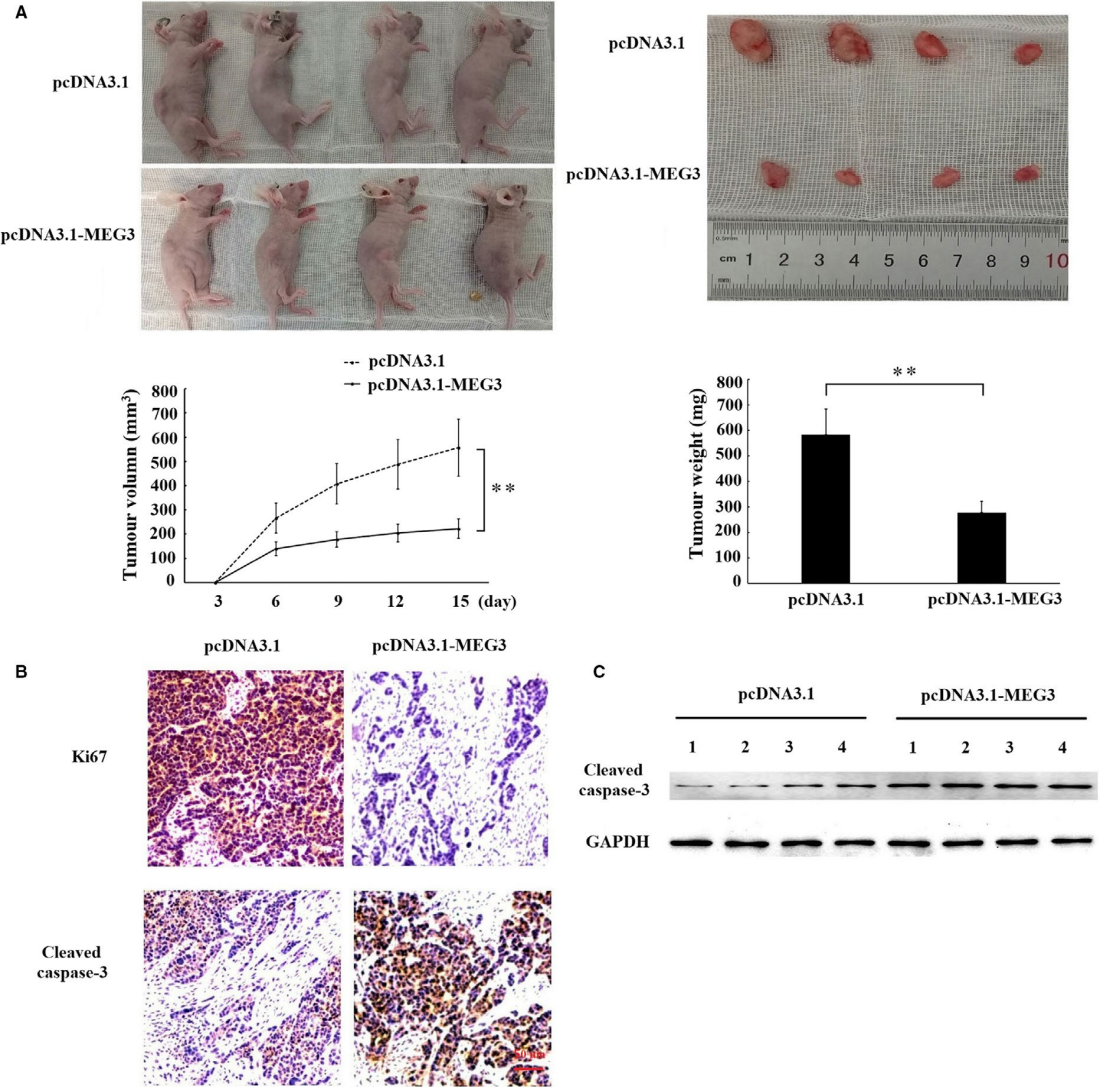
The effects of MEG3 on the growth of laryngeal cancer cells in vivo. Hep‐2 cells transfected with empty vector or MEG3 plasmid were injected into nude mice (n = 4/group). A, Tumour volumes were measured every 3 d after injection. At 15 d after injection, xenograft tumours were harvested and weighed. B, Immunohistochemistry assay was performed to assess the protein levels of Ki67 and cleaved caspase‐3. Magnification: ×400, scale bars: 50 μm. C, Western blotting was employed to measure protein level of cleaved caspase‐3 in xenograft tumour tissues. The data were obtained from three independent experiments and presented as mean ± SEM, ** *P* < .01

We further investigated the effect of MEG3 on the growth, proliferation and apoptosis of laryngeal cancer xenograft tumour in nude mice. The results showed that the ratio of tumour growth was decreased in pcDNA3.1‐MEG3 group as evidenced by a notable decrease in tumour weight and volume (Figure 4A). Moreover, the IHC results showed that Ki67 protein expression, a cellular marker for proliferation, was also decreased in pcDNA3.1‐MEG3 group (Figure 4B). Meanwhile, the expression of cleaved caspase‐3, a marker for apoptosis, was increased in pcDNA3.1‐MEG3 group measured by IHC and Western blotting (Figure 4B,C). Taken together, these results suggest that MEG3 inhibits the growth and proliferation and induces the apoptosis of laryngeal cancer cells in vivo.

### 3.4 Reciprocal repression of miR‐23a and MEG3

**Figure 5:**
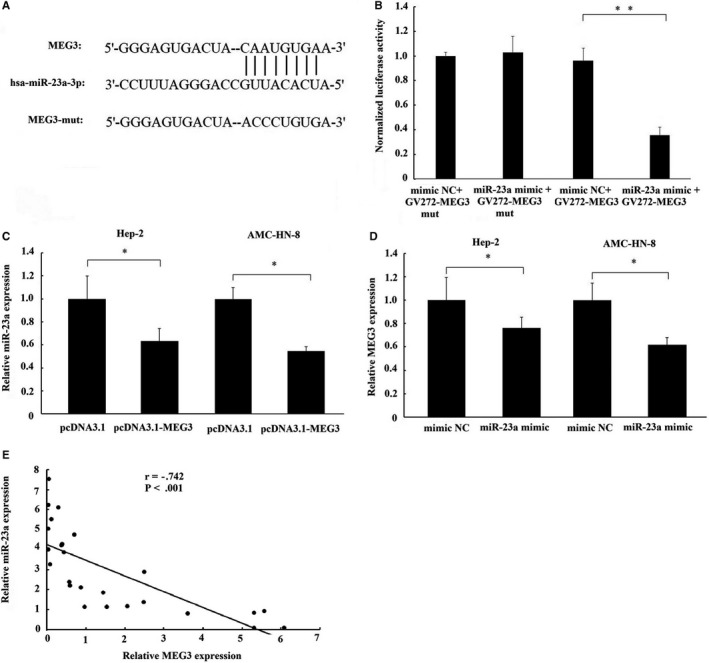
The interaction of MEG3 with miR‐23a. A, The binding site of miR‐23a on MEG3 transcript. B, Luciferase reporter gene assay validates the interaction between MEG3 and miR‐23a in Hep‐2 cells. GV272‐MEG3, luciferase vector of MEG3; GV272‐MEG3‐mut, MEG3 mutant vector. C, The effect of MEG3 overexpression on miR‐23a expression in Hep‐2 and AMC‐HN‐8 cells. D, The effect of miR‐23a overexpression on MEG3 expression in Hep‐2 and AMC‐HN‐8 cells. E, A negative relationship between MEG3 and miR‐23a expression existed in laryngeal cancer tissues. The data were presented as mean ± SEM, *P < .05, **P < .01

To confirm whether miR‐23a binds to MEG3 specifically, a luciferase reporter assay was employed. The result of luciferase reporter assay showed that cotransfection with miR‐23a mimic and MEG3 luciferase reporter significantly decreased the luciferase activity in Hep‐2 cells as compared with cotransfection with mimic NC and MEG3 luciferase reporter; meanwhile, the luciferase activity did not decrease by cotransfection with miR‐23a mimic and MEG3 mutant luciferase reporter (Figure 5B). This result provided a direct evidence that miR‐23a binds to MEG3 specifically. Moreover, we found that overexpression of MEG3 reduced miR‐23a expression in Hep‐2 and AMC‐HN‐8 cells (Figure 5C). Conversely, overexpression of miR‐23a could also decrease MEG3 expression in Hep‐2 and AMC‐HN‐8 cells (Figure 5D). In addition, MEG3 expression negatively correlated with miR‐23a level in laryngeal cancer samples (Figure 5E). All these data demonstrate that a reciprocal negative regulation exists between miR‐23a and MEG3.

### 3.5 MEG3 regulated APAF‐1 expression via miR‐23a

**Figure 6:**
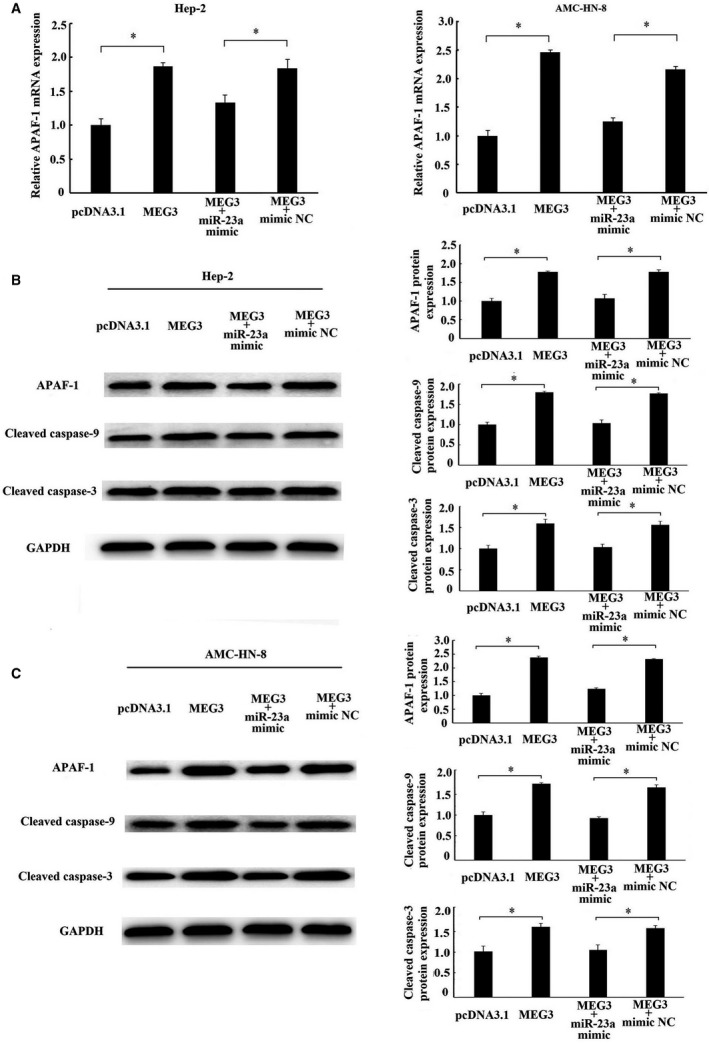
Regulation of APAF‐1 by MEG3 was mediated by miR‐23a. MEG3 plasmid (pcDNA3.1‐MEG3) and empty vector (pcDNA3.1) alone or together with miR‐23a mimics or miR‐23a‐negative control (mimic NC) were transfected into Hep‐2 and AMC‐HN‐8 cells. A, The mRNA expression of APAF‐1 in Hep‐2 and AMC‐HN‐8 cells was detected by RT‐qPCR. Western blotting analysed the protein levels of APAF‐1, cleaved caspase‐9 and cleaved caspase‐3 in Hep‐2 (B) and AMC‐HN‐8 cells (C). The data were obtained from three independent experiments and presented as mean ± SEM. * *P* < .05

As APAF‐1 is a target of miR‐23a^16^ and miR‐23a has be demonstrated to bind to MEG3 specifically through luciferase reporter assay, the present study have been suggested that MEG3 regulated APAF‐1 expression via sponging miR‐23a. As expected, overexpression of MEG3 increased the mRNA (Figure 6A) and protein expression of APAF‐1, and subsequently led to an increase in the protein level of cleaved caspase‐9 and cleaved caspase‐3 in Hep‐2 (Figure 6B) and AMC‐HN‐8 cells (Figure 6C). However, the activation of APAF‐1, cleaved caspase‐9 and cleaved caspase‐3 by MEG3 was reversed by miR‐23a overexpression (cotransfection with MEG3 and miR‐23a mimic). These data suggest that the regulation of APAF‐1 by MEG3 is mediated by miR‐23a.

## 4 DISCUSSION

Maternally expressed gene 3 is an imprinted gene commonly expressed in several normal tissues, such as pituitary and cerebellum.^11^ However, it is usually down‐regulated in non–small‐cell lung cancer,^17^ breast cancer,^18^ hepatocellular carcinoma,^19^ gastric cancer,^14^ cervical cancer,^20^ oesophageal cancer,^21^ prostate cancer,^22^ nasopharyngeal carcinoma^23^ and gliomas,^24^ even lost in pituitary tumours, meningiomas and myelomas.^25^ The low expression of MEG3 in cancer cells and tissues is likely to be related to aberrant promoter hypermethylation.^21^, ^25^ Like most of previous researches, we found that the expression of MEG3 was generally down‐regulated in laryngeal cancer tissues. Moreover, the decreased expression of MEG3 was associated with advanced clinical stage, which was similar to Zhuo et al finding in breast cancer^19^ and Tian et al finding in osteosarcoma.^26^ Taken together, our findings suggest that MEG3 may serve as a novel biomarker for the progress of laryngeal cancer.

Maternally expressed gene 3 is initially found to be a growth suppressor in human cancer cells including HeLa, MCF‐7 and H4.^11^ Growing evidence has also demonstrated that MEG3 characterizes as a tumour suppressor in different types of cancer as evidence that overexpression of MEG3 could suppress the proliferation and induce apoptosis of cancer cells.^14^, ^17^, ^18^, ^19^, ^20^, ^21^, ^22^, ^23^, ^24^, ^25^ However, little is known about the role of MEG3 in laryngeal cancer up to date. In the present study, we found that overexpression of MEG3 inhibited the proliferation and induced the apoptosis in human laryngeal cancer cells and the similar evidence was also provided in vivo. These results were consistent with previous findings in non–small‐cell lung cancer^17^ and prostate cancer,^22^ and further suggest that MEG3 may serve as a novel tumour suppressive LncRNA to regulate laryngeal carcinogenesis.

Recent studies have illuminated that LncRNAs can function as a kind of ceRNA to interact with miRNAs and further regulate the expression of target mRNAs through sharing miRNA response elements (MREs).^27^ Thus, LncRNA‐miRNA‐mRNA forms a novel regulatory network at post‐transcription level.^28^ Considering that our previous studies have demonstrated that miR‐23a is up‐regulated and characterized an oncogene in laryngeal cancer, we firstly explored the possibility for the interaction between MEG3 and miR‐23a. The result of luciferase report gene confirmed that miR‐23a could bind to MEG3 specifically. Interestingly, we found that overexpression of MEG3 repressed miR‐23 expression and vice versa. According to this result, we inferred that there existed reciprocal repression between miR‐23a and MEG3 in laryngeal cancer. Similarly, Braconi *et al* reported that MEG3 and miR‐29a formed a reciprocal regulatory loop in hepatocellular cancer.^29^ Also, there was a reciprocal repression between MEG3 and miR‐34a in the process of hepatic ischaemia reperfusion.^30^ The recent studies have demonstrated that the reciprocal repression between LncRNA and miRNA displays in an Argonaute 2 (Ago2)‐dependent manner and implies that both LncRNA and miRNA might be in the RNA‐induced silencing complex (RISC).^30^, ^31^ Therefore, we inferred that this reciprocal regulation of MEG3 and miR‐23a is likely at the post‐transcriptional level involving the RISC.

Apoptotic protease activating factor‐1, a tumour suppressor, is a major target of miR‐23a verified in various types of cancer, including laryngeal cancer.^16^, ^32^, ^33^ In this study, we found that overexpression of MEG3 remarkably increased APAF‐1 expression. Several studies have demonstrated that APAF‐1 is the adaptor molecule which in the presence of cytosolic cytochrome c and dATP interacts with pro‐caspase‐9, resulting in the sequential cleavage and activity of caspase‐9 and caspase‐3, followed by apoptosis.^34^, ^35^ Perkins C et al have further demonstrated that overexpression of APAF‐1 promoted the oligomerization of pro‐caspase‐9, thereby enhancing the cleavage and activation of caspase‐9 and caspase‐3.^36^ Our data also support this by showing that the levels of cleaved caspase‐9 and caspase‐3 were enhanced along with an increasing in APAF‐1 expression by MEG3 overexpression. These results imply that MEG3 can activate mitochondrial apoptosis‐related pathway, and at least in part explain why MEG3 can induce apoptosis and inhibit the growth and proliferation of laryngeal cancer cells. Meanwhile, we found that the activations of APAF‐1 and its downstream caspases induced by MEG3 were rescued by overexpression of miR‐23a, which implicated that the regulation of APAF‐1 by MEG3 is mediated by miR‐23a. Taken together, these results support the conclusion that MEG3, miR‐23a and APAF‐1 form a ceRNA regulatory network in the progression of laryngeal cancer.

However, we must acknowledge that there existed two limitations in this study. First, although we have already demonstrated that the activations of APAF‐1 and its downstream caspases induced by MEG3 were rescued by miR‐23a, it is still not known whether the phenotype in laryngeal carcinoma induced by MEG3 was rescued by miR‐23a. Thus, more studies should be conducted to confirm what extent the phenotype in laryngeal cancer induced by MEG3 was rescued by miR‐23a. Second, besides APAF‐1, miR‐23a is found to target many apoptosis‐related genes, such as PDCD4,^37^ BCL2^38^ and Fas.^39^ Therefore, there exists the possibility that the apoptosis rescued by miR‐23a overexpression may attribute to the regulation of these apoptosis‐related genes. However, there is not yet enough evidence in our study to confirm the possibility and we must acknowledge that this is also a limitation of our present study.

In conclusion, the present study demonstrates that MEG3 acts as a novel tumour suppressive LncRNA in laryngeal cancer for the first time. Furthermore, MEG3 acts as a ceRNA to regulate APAF‐1 expression via competitively binding to miR‐23a, thereby regulating the progression of laryngeal cancer. Undoubtedly, identifying the role and mechanism of MEG3 in laryngeal cancer will bring a novel insight into the progression of laryngeal cancer.

## CONFLICT OF INTEREST

The authors have no conflict of interest.

## AUTHOR CONTRIBUTIONS

Xiaowen Zhang and Zhijie Li designed experiments. Xiaowen Zhang, Nan Wu and Jin Wang performed experiments. Xiaowen Zhang and Nan Wu wrote the manuscript. Xiaowen Zhang and Jin Wanganalysed data.
